# Attention and multisensory integration of emotions in schizophrenia

**DOI:** 10.3389/fnhum.2013.00674

**Published:** 2013-10-18

**Authors:** Mikhail Zvyagintsev, Carmen Parisi, Natalia Chechko, Andrey R. Nikolaev, Klaus Mathiak

**Affiliations:** ^1^Department of Psychiatry, Psychotherapy and Psychosomatics, Medical School, RWTH Aachen UniversityAachen, Germany; ^2^IZKF Aachen, RWTH Aachen UniversityAachen, Germany; ^3^JARA – Translational Brain MedicineAachen, Germany; ^4^Laboratory for Perceptual Dynamics, University of LeuvenLeuven, Belgium

**Keywords:** schizophrenia, attention, multisensory integration, emotions, interference

## Abstract

The impairment of multisensory integration in schizophrenia is often explained by deficits of attentional selection. Emotion perception, however, does not always depend on attention because affective stimuli can capture attention automatically. In our study, we specify the role of attention in the multisensory perception of emotional stimuli in schizophrenia. We evaluated attention by interference between conflicting auditory and visual information in two multisensory paradigms in patients with schizophrenia and healthy participants. In the first paradigm, interference occurred between physical features of the dynamic auditory and visual stimuli. In the second paradigm, interference occurred between the emotional content of the auditory and visual stimuli, namely fearful and sad emotions. In patients with schizophrenia, the interference effect was observed in both paradigms. In contrast, in healthy participants, the interference occurred in the emotional paradigm only. These findings indicate that the information leakage between different modalities in patients with schizophrenia occurs at the perceptual level, which is intact in healthy participants. However, healthy participants can have problems with the separation of fearful and sad emotions similar to those of patients with schizophrenia.

## Introduction

Schizophrenia is a severe mental disorder characterized by impairments in a wide spectrum of psychological functions. Eight separable cognitive domains represent essential deficits in schizophrenia: speed of processing, attention/vigilance, working memory, verbal learning and memory, visual learning and memory, reasoning and problem solving, verbal comprehension, and social cognition (Nuechterlein et al., 2004). Among these domains, the deficit of attention stands out because the selection of relevant information is crucial for any perceptual or cognitive function. Correspondingly, the attentional deficit itself may cause impairment in other domains. This view has been held from the beginning of twentieth century, when Eugen Bleuler (1911) proposed that most schizophrenia deficits originate from the fundamental deficit of attention.

Contrary to the well-established role of attention in the perception of physical stimuli (Posner and Peterson, 1990), findings on the effect of attention on perception of emotions are inconsistent. Some works showed that the perception of emotions is automatic (Vuilleumier et al., 2001; Pessoa and Ungerleider, 2004; Vuilleumier, 2005), whereas other studies have demonstrated that attention contributes to the selection of emotional stimuli (Pessoa et al., 2002, 2005; Erthal et al., 2005; Mitchell et al., 2007; Lim and Pessoa, 2008). Emotions are severely affected in schizophrenia. Unpredictable or inappropriate emotional responses and anhedonia are typical clinical features of the disease. In fact, all aspects of emotional behavior, such as emotion expression, experience, and recognition, are impaired in schizophrenia (Trémeau, 2006). Specifying the effect of attention on emotional behavior in patients with schizophrenia is important for the development of diagnostics and treatment.

The aim of our study is to specify the role of attention in the perception of emotions in schizophrenia. Because of the limited processing resources, the attentional selection of relevant information is crucial for perception. The role of attention in perception can be estimated by measuring a conflict that occurs between incongruent features of the same stimulus. Consequently, the most common approach to test selective attention is based on the interference effect. For example, in the classical Stroop task (Stroop, 1935), participants are presented with words written with inks of different colors and their task is either to read a word ignoring a color or to name a color ignoring a word. When the name of the color corresponds to the ink color, the participants' responses are facilitated. The Stroop interference has been widely used as a measure of attention for studying perceptual encoding, processing, and decision in healthy people (reviewed in MacLeod, 1991). Patients with schizophrenia show significantly stronger Stroop interference compared to healthy participants, suggesting the presence of attentional deficit (Perlstein et al., 1998; Barch et al., 1999, see Henik and Salo, 2004 for review).

In our study, we were interested in cases in which emotional stimuli come from different sensory modalities. Impairment of multisensory integration is a well-known problem of schizophrenia (Ross et al., 2007; Szycik et al., 2009; Seubert et al., 2010; Williams et al., 2010), which can be explained by an attention deficit (de Jong et al., 2010). Early studies in healthy people did not find any effect of attention on multisensory integration (reviewed in De Gelder and Bertelson, 2003), but recent works indicate the specific role of attention in multisensory perception (Talsma et al., 2010; Zvyagintsev et al., 2011; Roudaia et al., 2013). Consequently, we will focus on the role of attention in the multisensory perception of emotional stimuli in patients with schizophrenia.

To study attention in multisensory integration, the Stroop task can be modified so that the interfering stimuli arrive from different modalities (Zvyagintsev et al., 2009; Klasen et al., 2011). If the stimuli from different modalities interfere in their emotional content, the interference effect can be used as a tool for studying the multisensory integration of emotional information. For example, de Gelder et al. (2005) investigated the categorization of happy and sad facial expressions presented together with voices with happy and sad prosody. The interference effect from *auditory* incongruent stimuli was weaker in patients with schizophrenia than in healthy participants. The authors explained this finding by impaired cross-modal integration of the emotional stimuli in schizophrenia. In the second experiment, the authors investigated the categorization of the voices with happy and sad prosody in the presence of emotionally congruent and incongruent faces. It was found that the interference effect from *visual* incongruent stimuli was higher in patients with schizophrenia than in healthy participants. To explain the inconsistency between the first and second experiments, the authors assumed that cross-modal interference in schizophrenia depends on the target modality: hypo-integration of the auditory incongruent stimuli and hyper-integration of the visual incongruent stimuli may occur because of general visual dominance in audiovisual perception. However, in another work, the same authors found weaker interference from the *visual* incongruent stimuli in patients with schizophrenia than in healthy participants in the categorization of voices with fear and happy prosody (de Jong et al., 2009).

The inconsistency of these results conceals the factors that contribute to impairment of multisensory integration in schizophrenia, particularly the role of attention. In the present study, we used the multimodal interference effect as a measure of attention in the perception of emotional stimuli coming from different modalities. Similar to de Gelder et al. (2005), we examined the emotional interference of auditory and visual stimuli in patients with schizophrenia. However, instead of happy and sad emotions, we used fearful and sad emotions. We chose these emotions because the categorization of the fearful facial expression suffers most in patients with schizophrenia compared with healthy participants (Kohler et al., 2003; Johnston et al., 2006; Schneider et al., 2006; Habel et al., 2010, see also Morris et al., 2009 for a review). In addition, the categorization of sad faces is also impaired in schizophrenia (Johnston et al., 2006; Habel et al., 2010), and the misattribution of sad and fearful faces is the highest among other facial expressions for healthy participants (Johnston et al., 2006; Habel et al., 2010). These observations suggest that fearful and sad emotions may be more likely to be confused than happy and sad emotions, making the task demanding even for healthy participants.

We used two multisensory paradigms in which congruency of the auditory and visual information was manipulated when participants were categorizing the visual stimuli. In one paradigm, interference occurred in the spatiotemporal properties of the dynamic auditory and visual stimuli. In another paradigm, interference occurred in the emotional content of the auditory and visual stimuli. Here, participants were asked to categorize sad and fearful facial expressions while listening to the pseudowords with sad and fearful prosody. We assessed the interference effects in two paradigms for patients with schizophrenia compared to healthy participants. We hypothesized that because of attentional deficit in schizophrenia, the interference effects in patients should occur in both paradigms. However, healthy participants may be able to avoid the information leakage between modalities and accurately select the target stimuli.

## Methods

### Participants

Twenty patients with schizophrenia and twenty healthy participants took part in the study. Healthy participants were recruited via public advertisement. They had normal or corrected to normal vision, normal hearing, and no history of neurological comorbidity, psychiatric illness, and psychopharmacological therapy. Patients were recruited among inpatients in the Clinic for Psychiatry, Psychotherapy and Psychosomatics, RWTH Aachen University Hospital, Germany. The diagnosis of schizophrenia was made by the treating physician according to the ICD-10. Symptoms were assessed with the Positive and Negative Symptom Scale (PANSS) by an experienced neuropsychologist. All patients received the second-generation antipsychotic medication with 66 ± 28% of the daily defined maximal dose (DDD, WHO Collaborating Centre for Drug Statistics Methodology, 2012). Five patients were additionally taking anti-depressive medication (DDD = 39 ± 37%). The groups were matched for gender, age and parental education. The sociodemographic and illness-related characteristics of participants are listed in the Table 1. Participants of both groups received 10 € for their participation. The study was conducted in accordance with the Declaration of Helsinki, and the protocol was approved by the Ethics Committee of the RWTH Aachen University. Written informed consent was obtained from all participants following a complete description of the study and all experimental procedures.

**Table 1:** **Sociodemographic characteristics of the groups and the illness-related data for the patients**.

	**Patients, *n* = 15 (mean ± *SD*)**	**Controls, *n* = 18 (mean ± *SD*)**	**Two-sample *t*-test, *df* = 32 (*p*)**
Age, years	42.9 ± 7.7	42.5 ± 7.1	0.1 (0.9)
Gender	13 m, 7 f	13 m, 7 f	0 (1)
Education, years	12.0 ± 1.4	11.4 ± 1.8	1.3 (0.2)
Parental Education, years	9.9 ± 1.7	10.0 ± 1.7	0.1 (0.9)
Disease duration, years	15.7 ± 8.7	−	−
PANSS positive, score	11.4 ± 3.9	−	−
PANSS negative, score	13.1 ± 4.0	−	−
PANSS general, score	26.2 ± 5.6	−	−
PANSS total, score	50.5 ± 12.0	−	−

### Stimuli for the looming paradigm

We used two paradigms that will be referred to as LOOMING and FACE.

**Figure 1:**
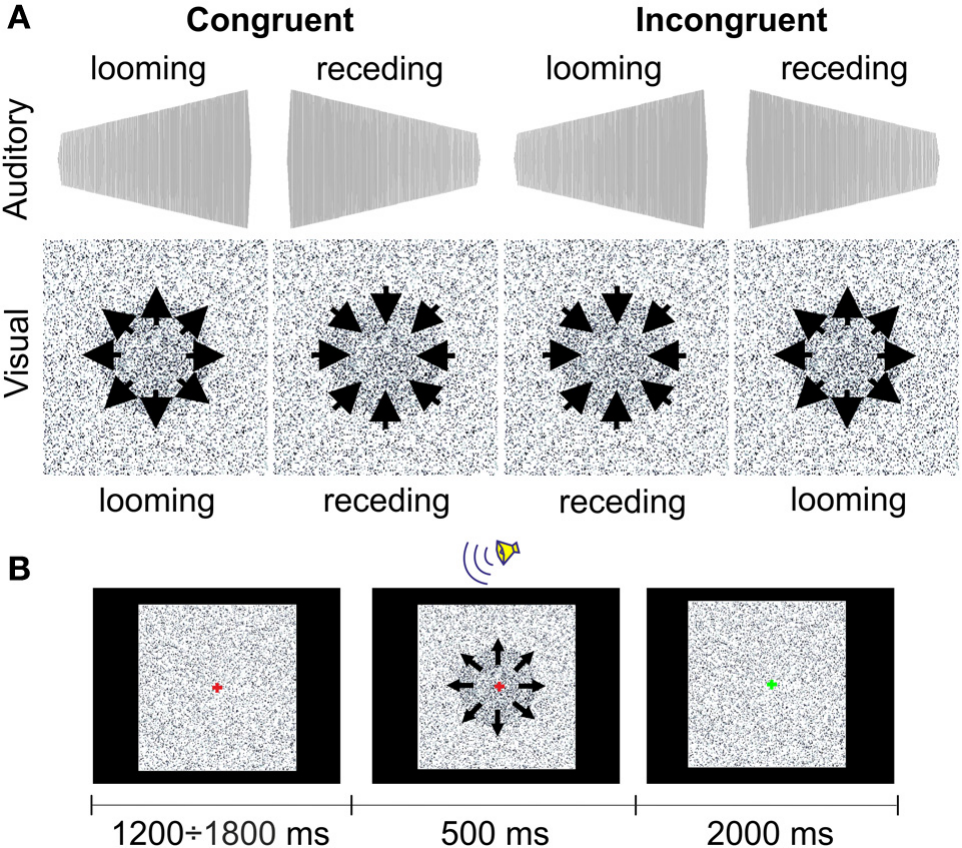
**The stimuli (A) and the procedure (B) in the LOOMING paradigm**.

The LOOMING paradigm included two audiovisually congruent and two incongruent conditions (Figure 1A). The looming and receding sounds were the 500-ms sine waves that linearly rose or fell in intensity with initial and terminal intensities of 42(57) and 57(42) dB, respectively. A recent study performed by Bach et al. (2011) showed that at these intensities, patients with schizophrenia do not differ from healthy participants in accuracy of the sound direction detection (e.g., receding or looming). Both sounds had an initial falling/raising time of 10 ms. The auditory stimuli were prepared using Csounds 5.09 Software (www.csounds.com). They were delivered binaurally via Sennheiser HD 600 headphones (Sennheiser Electronics Corp., CT). The visual stimuli were prepared using Presentation 7.0 Software (Neurobehavioral Systems, Inc., Albany, California; www.neurobs.com). Therefore, we created a matrix of 200 × 200 quadrants on the laptop screen. Each quadrant represented a 4 × 4 matrix of the display's pixels using a standard resolution of 1280 × 1024 pixels. The color of each quadrant was randomly assigned to the standard RGB gray scale values in the range from 0 (white) to 255 (black). During the entire session, the quadrants randomly changed their color intensity within this range with an interval of 16.7 ms. This was perceived as a twinkling of the background. The purpose of this twinkling was to make detection of the circle size changes (see below) more difficult. In the center of the matrix, we created a circle by increasing the color intensity of the quadrants, forming a filled circular shape by 10%. This circle either increased or decreased in size from 8 to 17° (looming condition) or from 17 to 8° (receding condition). The onsets of the circle appearance and the sound stimulus were synchronized with Presentation 7.0 software.

### Stimuli for the face paradigm

**Figure 2:**
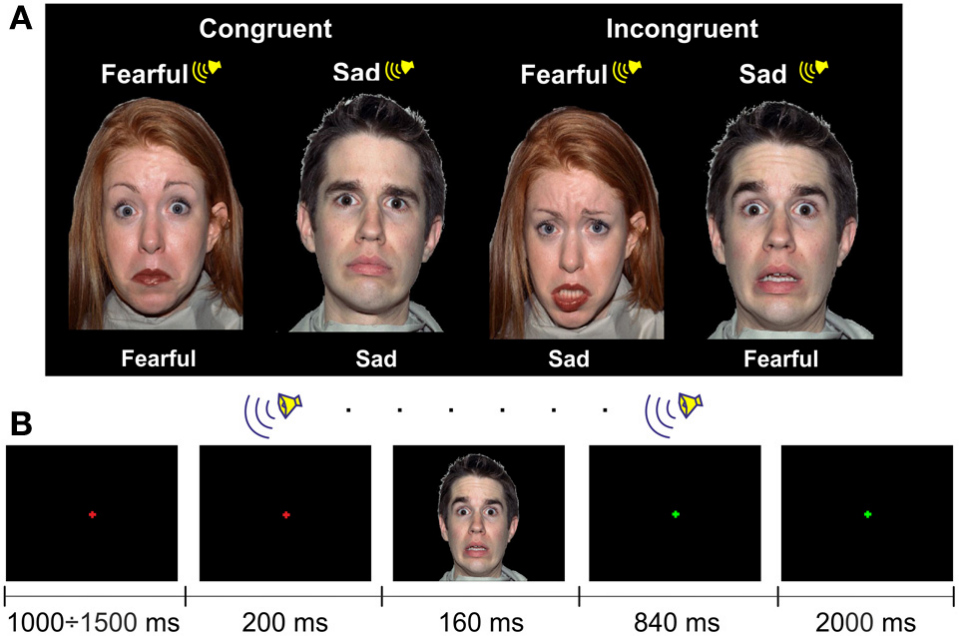
**The examples of the stimuli (A) and the procedure (B) in the FACE paradigm**.

The FACE paradigm included two audiovisual congruent and two audiovisual incongruent conditions (Figure 2A). However, here the congruency was related to the emotional content of the stimuli. For the visual stimuli, we selected 60 faces of 13 male and 13 female actors presenting an equal number of fearful and sad facial expressions from the NimStim Face Stimulus Set (Tottenham et al., 2009). The NimStim stimuli included faces with open and closed mouths. We chose an equal number of open- and closed-mouth faces and counterbalanced them between the conditions.

One face subtended ~12 × 16° of a visual angle (width × height) with a viewing distance of ~60 cm. The faces were presented on a black background.

Auditory stimuli were emotional pseudowords from the in-house dataset. They consisted of 84 sound files that contained 7 pseudowords pronounced by 3 male and 3 female actors with fear and sad prosodies (see examples of the stimuli in Supplementary Material).

The visual and auditory stimuli were presented on a laptop using Presentation 7.0 Software. The auditory stimuli were delivered binaurally via Sennheiser HD 600 headphones at 55 dB SPL.

### Experimental procedure

To evaluate attentional impairment, before the experiment, all participants underwent neuropsychological testing, which included the *Trail Making Test* A and B (TMT-A, TMT-B), the vocabulary test [*Wortschatztest* (*WST*)], and the *forward* and *backward digit span* tests [WST and digit span were taken from the German version of the *Wechsler Adult Intelligence Scale (WAIS-R)*, (Tewes, 1991); Table 2]. The TMT-A, -B aimed to test attention and processing speed, WST aimed to test verbal intelligence, and the digit span test aimed to test attention and working memory (Mesholam-Gately et al., 2009). In addition, symptoms of patients with schizophrenia were assessed with the positive and negative syndrome scale (PANSS; Table 1).

**Table 2:** **Neuropsychological assessment and comparison of healthy participants and patients with schizophrenia**.

**Test**	**Patients (mean ± *SD*)**	**Controls (mean ± *SD*)**	**Two-sample *t*-test, *df* = 32**
TMT-A, seconds	31.6 ± 11.5	23.1 ± 8.3	2.7 (0.01)^*^
TMT-B, seconds	79.4 ± 38.1	50.0 ± 13.2	3.3 (0.01)^*^
WST, items	29.2 ± 6.5	34.1 ± 3.6	2.9 (0.01)^*^
Digit span forward, items	7.5 ± 2.2	8.4 ± 2.5	1.2
Digit span backward, items	5.8 ± 2.1	7.0 ± 2.3	0.7

* p < 0.05.

In the LOOMING paradigm, 60 congruent trials with either audiovisual looming or receding stimuli and 60 incongruent trials were presented to each participant. In a looming congruent trial, the auditory stimulus increased in loudness and the visual stimulus increased in size. In a receding congruent trial, the auditory stimulus decreased in loudness and the visual stimulus decreased in size. In incongruent trials, the directions of the changes for the auditory and visual stimuli were opposite. Altogether, 120 trials were presented in random order to each participant.

Participants were instructed to look at the fixation cross of 0.1° of the visual angle centered at the screen. A trial started with a change of the color of the cross from green to red. Then, after a delay (which varied randomly between 1200 and 1800 ms), an audiovisual stimulus was presented for 500 ms (Figure 1B). After offset of the audiovisual stimulus, a green fixation cross was shown for 2000 ms. The task for the participants was to recognize the type of the visual stimulus. After the onset of the audiovisual stimulus, the participants had to press one of two buttons, which indicated the looming or receding type of the *visual* stimulus. Participants were instructed to answer as precisely and as quickly as possible. The response time was limited to 2000 ms after the offset of the audiovisual stimulus. The response buttons were counterbalanced between participants. The inter-trial interval varied randomly between 3700 to 4300 ms. The experiment lasted ~8 min.

In the FACE paradigm, 60 congruent trials with either sad or fearful emotional content and 60 incongruent trials were presented to each participant. In the congruent trials, the emotional content of the auditory and visual stimuli was matched, and in the incongruent trials, the emotional content was different. The face and the voice were always matched in gender, but a matched auditory stimulus was chosen randomly for a particular visual stimulus. The sequence of 120 trials was randomized for each participant.

Participants were instructed to look at the fixation cross of 0.1° of the visual angle centered at the screen. A trial started with a change of the color of the cross from green to red. Then, after a delay (which varied randomly between 1000 and 1500 ms), a pseudoword with a duration of 1000 ms was presented. In 200 ms after the pseudoword onset, a face was presented for 160 ms (Figure 2). After the offset of the visual stimulus, a green fixation cross was presented for 2840 ms, indicating to participants the response interval. The task was to categorize the facial expression: the participants had to press one of two buttons, which indicated sad or fearful emotion. Participants were instructed to answer as precisely and as quickly as possible. The response time was limited to 3000 ms after the onset of the visual stimulus, i.e., until the fixation cross changed the color. The type of the response button was counterbalanced between participants. The inter-trial interval varied randomly between 4200 to 4700 ms. The experiment lasted ~9 min.

The order of application of the LOOMING and FACE paradigms was counterbalanced between participants.

### Statistical analysis

For each participant and each paradigm, we considered the following variables:

1. the percentage of the trials with responses, regardless of condition and response correctness, as a ratio of the trials with responses to all trials;
2. the percentage of the trials with correct responses, regardless of condition, as a ratio of correct responses to all responses;
3. the average response time for all trials with responses, regardless of correctness of the response;
4. the percentage of the trials with correct responses for each condition as a ratio of correct responses to all responses for this condition (further referred as an accuracy rate).

To verify that both groups of participants attended to each paradigm and followed the instructions, we compared the percentage of the trials with responses between groups using a two-sample *t*-test for each paradigm separately.

We then tested the overall correctness of responses between groups by comparing the percentage of the trials with correct responses regardless of condition using a two-sample *t*-test for each paradigm separately.

Next, we compared the average response time for all trials with a response (regardless of its correctness) between the groups using a two-sample *t*-test for each paradigm separately.

The accuracy rates were averaged across stimulus conditions for congruent and incongruent trials in each paradigm. The accuracy rates were submitted to a repeated-measures ANOVA for each paradigm separately. In ANOVA, we used the within-factor of Congruency (congruent vs. incongruent) and the between-factor of Group (patients with schizophrenia vs. healthy participants). Whenever ANOVA revealed an interaction, we proceeded with the *post-hoc* LSD test.

The statistical analysis was performed with STATISTICA 10.0 software (StatSoft, Inc., Tulsa, OK).

## Results

One patient did not finish the study, and two patients and two healthy participants responded in less than 70% of trials in one of the paradigms. Two patients responded at the chance level in both congruent conditions (53 and 57% of correct answers) in one of the paradigms. These participants were excluded from further analyses. The remaining participants in both groups were the same for both paradigms.

The results of neuropsychological assessment and comparison between the groups are reported in the Table 2. Patients were significantly slower than healthy participants in the TMT-A and TMT-B and made significantly more errors in the WST. Although the digit span tests revealed the lower performance in the schizophrenia group than in controls, the differences between the groups in these tests were insignificant. The results of neuropsychological testing suggest that in our study, patients with schizophrenia had a moderate impairment of attention and a lower verbal IQ level than healthy participants.

We did not observe any difference between groups in the total number of responses in both paradigms: LOOMING [*t*_(31)_ = 0.9, *p* = 0.4] and FACE [*t*_(31)_ = 0.7, *p* = 0.5; Table 3]. This suggests that participants of both groups followed the instructions and were attentive. Further, we did not find any difference between groups in the average response time for the trials with responses: LOOMING [*t*_(31)_ = 0.6, *p* = 0.7] and FACE [*t*_(31)_ = 0.8, *p* = 0.4].

**Table 3:** **Overall performance of participants in two paradigms**.

**Paradigm**	**Parameter**	**Patients (mean ± *SE*)**	**Controls (mean ± *SE*)**	**Two-sample *t*-test, *df* = 32**
LOOMING	Total, %	94.2 ± 1.6	96.4 ± 1.2	0.9
	Correct, %	76.2 ± 3.8	85.0 ± 2.0	2.1 (0.04)^*^
	Response time, ms	1208 ± 36	1235 ± 40	0.6
FACE	Total, %	99.3 ± 0.3	99.7 ± 0.1	0.7
	Correct, %	72.4 ± 1.9	84.8 ± 1.5	5.1 (0.001)^**^
	Response time, ms	1250 ± 56	1280 ± 44	0.8

* p < 0.05,

** p < 0.01.

However, the difference between groups was observed in the accuracy rate in both paradigms (Table 3). Therefore, we submitted this measure to ANOVAs for each paradigm separately.

In the LOOMING paradigm, the ANOVA revealed a significant effect of Congruency *F*_(1, 31)_ = 6.3, *p* = 0.01 and a Congruency × Group interaction: *F*_(1, 31)_ = 4.7, *p* = 0.03, but no effect of Group [*F*_(1, 31)_ = 3.5, *p* = 0.08]. The *post-hoc* test showed that the effect of Congruency was significant only in the patients' group (*p* < 0.01), but not in the healthy participants' group (*p* = 0.8).

**Figure 3:**
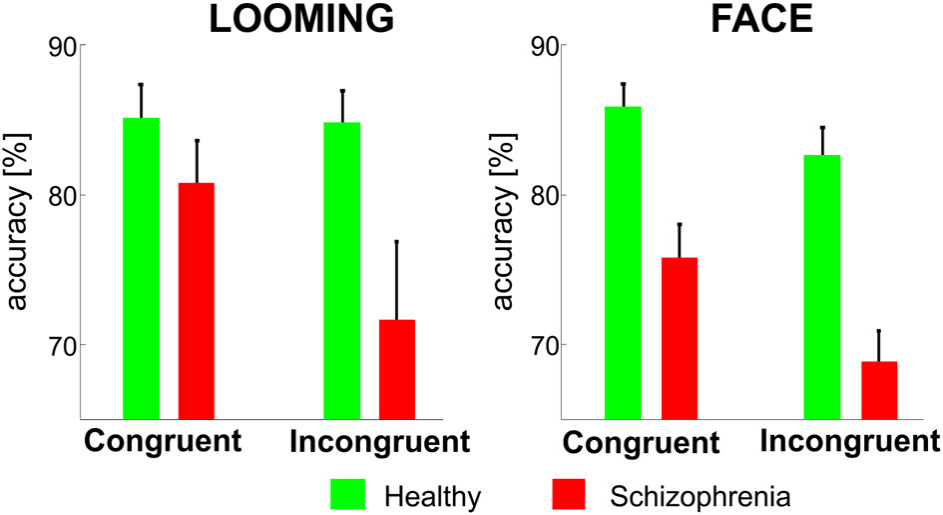
**The mean accuracy rates (±SE) for the congruent and incongruent conditions in the LOOMING paradigm (left panel) and in the FACE paradigm (right panel) for healthy participants and patients with schizophrenia**. In LOOMING, the difference between congruent and incongruent conditions exists only in patients with schizophrenia. In FACE, the difference between the congruent and incongruent conditions exists in both groups.

In the FACE paradigm, the ANOVA revealed a significant effect of Congruency: *F*_(1, 31)_ = 24.3, *p* < 0.001 and Group: *F*_(1, 31)_ = 23.2, *p* < 0.001 and no Congruency × Group interaction [*F*_(1, 31)_ = 3.1, *p* = 0.1] (Figure 3).

## Discussion

The present study examined the effect of attention on the categorization of visual stimuli in two multisensory paradigms in patients with schizophrenia and healthy controls. Attention was measured as an interference effect between conflicting auditory and visual information. In the first (LOOMING) paradigm, the interference occurred between *dynamic physical features* of the auditory and visual stimuli, i.e., it occurred at the perceptual level. In the second (FACE) paradigm, the interference occurred between the *emotional content* of the auditory and visual stimuli.

In the perceptual (LOOMING) paradigm, the interference was observed in patients with schizophrenia, but not in healthy participants. In other words, the incongruent auditory input had an impact on visual stimuli categorization only in patients. Because interference in this paradigm occurred between physical features of the auditory and visual stimuli, this finding indicates a leakage of sensory information between the auditory and visual modalities. This suggests insufficiency of the attentional mechanism, which is responsible for the separation of relevant and irrelevant information flow in schizophrenia.

In the emotional (FACE) paradigm, the interference of auditory and visual stimuli occurred in both groups. Because interference in this paradigm occurred between the emotional contents of the audiovisual stimuli, this finding indicates facilitated the fusion of emotional information coming from the auditory and visual modalities, supporting the view that emotion perception is automatic (Vuilleumier et al., 2001; Pessoa and Ungerleider, 2004; Vuilleumier, 2005).

The results obtained in two paradigms cannot be compared directly because of several differences between them, e.g., different durations of the stimuli, response time windows and inter-trial intervals; different synchrony of the stimulus onsets; and different physical properties of the stimuli. However, we can compare the paradigms qualitatively and consider their consistency with the previous findings.

The results from the perceptual paradigm corroborate the previous studies based on the Stroop task, which showed higher interference between task-relevant and irrelevant information in patients with schizophrenia compared to healthy controls (Perlstein et al., 1998; Barch et al., 1999; Boucart et al., 1999, see Henik and Salo, 2004 for review). Our study extends this observation to multisensory perception: patients with schizophrenia have difficulties in concentrating on the task-relevant information, which comes not only from the same modality but also from different modalities.

In the emotional paradigm, the interference effects were similar for the healthy and schizophrenia groups. This observation is inconsistent with the view on the general impairment of the categorization of emotions in schizophrenia (Kohler et al., 2003; Johnston et al., 2006; Habel et al., 2010). It is also distinct from the observation that interference between auditory and visual emotional information in face categorization is lower in patients with schizophrenia compared to healthy participants (de Gelder et al., 2005). The latter finding was interpreted as evidence for the impairment of multisensory integration in schizophrenia. Our result can be explained by the types of emotional stimuli used. de Gelder et al. (2005) used happy and sad emotions, whereas we used fearful and sad emotions. Indeed, the common finding in schizophrenia studies was the impaired categorization of fearful facial expressions (Kohler et al., 2003; Johnston et al., 2006) and the mis-categorization of sad and fearful faces (Johnston et al., 2006). The significant effect of Group observed in our study indicates that patients had difficulties in the categorization of emotional faces even in the congruent condition, whereas de Gelder et al. (2005) did not observe differences in the categorization of the congruent stimuli between patients and healthy controls. Taken together, these observations suggest that the categorization task in our study was more difficult than in the study by de Gelder et al. (2005). This is a possible consequence of the larger similarity between fearful and sad emotions compared to happy and sad emotions. The similar emotional information can be easily confused because it competes for the same brain resources of recognition and categorization. Future research should include a wider range of tested emotions, perhaps including mixtures of emotion, to determine a difference in emotion confusion between patients and controls.

A possible limitation of our experimental design is the absence of the unimodal stimuli as a control condition. Although the multisensory interference is repeatedly reported (Talsma et al., 2010), without such a control, we cannot completely exclude the possibility that the patients had impairments of multisensory binding. Moreover, despite previous research suggesting that the second-generation antipsychotics have little influence on attention or even improve it in the long term (Bilder et al., 2002), we cannot completely exclude the effect of medication on the results of our experiment.

In sum, our results indicate that the deficit of attention in schizophrenia results in a mixture of the multimodal stimuli, which can be separated by healthy participants. This indicates that the fusion of task-relevant and irrelevant information that comes via the auditory and visual channels occurs at a relatively low perceptual level. This finding demonstrates how the fundamental deficit of attention in schizophrenia (Bleuler, 1911; Nuechterlein et al., 2004) may affect multisensory integration.

## Author contributions

Mikhail Zvyagintsev, Carmen Parisi, and Klaus Mathiak designed the study; Mikhail Zvyagintsev and Carmen Parisi prepared the protocol for the study; Carmen Parisi performed the data collection; Mikhail Zvyagintsev, Andrey R. Nikolaev and Carmen Parisi analyzed the data; Mikhail Zvyagintsev and Andrey R. Nikolaev wrote the manuscript; all authors contributed to and approved the final manuscript.

### Conflict of interest statement

The authors declare that the research was conducted in the absence of any commercial or financial relationships that could be construed as a potential conflict of interest.
